# Case Report: Epithelioid angiosarcoma of the pleura

**DOI:** 10.3389/fsurg.2024.1393159

**Published:** 2024-06-27

**Authors:** Niels Michael Dörr-Jerat, Claus May, Jürgen Knolle, Sylke Schmidt, Marcus Krüger

**Affiliations:** ^1^ Department of Thoracic Surgery, Martha—Maria Hospital Halle, Dölau, Germany; ^2^ Department of Pathology, Martha—Maria Hospital Halle, Dölau, Germany; ^3^ Department of Anesthesiology, Martha—Maria Hospital Halle, Dölau, Germany

**Keywords:** sarcoma, angiosarcoma, thoracic surgery, rare disease (RD), pleura

## Abstract

**Introduction:**

We present the case of a patient with recurrent bilateral hemothorax. After misdiagnosis despite several histological samples, a pleural manifestation of epithelioid angiosarcoma was diagnosed by further immunohistological staining. Based on this situation, we aim to sensitize the reader to this rare disease.

**Main concerns and important clinical findings:**

A 73-year-old fully conscious woman presented with dyspnea for 3 days. She was in stable general condition, pain was denied, she had a history of cigarette smoking, she had no cardiopulmonary events, and she was not receiving any anticoagulation medication. Physical examination revealed decreased breath sounds on the left side, and her hemoglobin level was 7.0 mmol/L.

**Primary diagnoses, interventions, and outcomes:**

The initial chest x-ray showed a left-sided effusion. Hemothorax was then diagnosed. Further investigation revealed no evidence of malignancy (CT, EBUS, cytology, etc.). VATS was performed, and biopsies of pleural lesions did not reveal congruent findings for the hemothorax. Due to recurrent bilateral hemothorax with the need for erythrocyte transfusion, the patient underwent several operations, including histological sampling, without evidence of malignancy. After further processing, an additional pathological report revealed an epithelioid angiosarcoma defined by massively proliferating epithelioid cells strongly positive for ERG and CD31 and negative for CD34. The neoplastic cells coexpressed D2-40 (podoplanin). Finally, due to multiple cerebral metastases, palliative therapy was indicated.

**Conclusion:**

Physicians and pathologists treating spontaneous hemothorax need to have broad knowledge of the possible, sometimes rare, etiologies. If the clinical course and intraoperative findings do not agree with the histopathological results, this finding must be questioned, and further immunohistochemical staining is mandatory. Thus, in the case of recurrent hemothorax, angiosarcoma of the pleura should also be considered for differential diagnosis.

## Introduction

Angiosarcomas account for 1%–2% of soft tissue sarcomas and arise from endothelial cells of small blood and lymphatic vessels and are commonly found in the skin, soft tissues, liver, spleen, heart and breast (1, 2). Thus, primary epithelioid angiosarcoma of the pleura is an extremely rare malignancy. Approximately 46 case reports have been published to date (3). In general, angiosarcoma often presents with benign symptoms, despite the presence of advanced disease with infiltrative growth and metastatic spread (4). In patients with a pleural origin, the most common symptoms are dyspnea, chest tightness, pain, pleural thickening, pleural effusion and recurrent hemothorax. Distinct mass formation is uncommon but has been described (5, 6). The clinical presentation may be confused with malignant mesothelioma or even benign diagnoses (7–9). The average age at presentation is 55 years, and men are more commonly affected than women are (6). Surgery is appropriate for localized tumors. However, the prognosis is poor. Although most patients die shortly after diagnosis, some authors argue that a multidisciplinary approach to treating angiosarcoma, consisting of surgery, radiotherapy and chemotherapy, may result in a positive outcome (4).

We present the unusual case of a 73-year-old female ex-smoker who presented to our emergency department on her own initiative with progressive dyspnea 3 days in duration and suspected pneumonia. Based on this situation, our aim is to sensitize the reader's clinical view to this extremely rare disease to avoid possible pitfalls. The patients' relatives were asked for their consent and agreed to publication.

## Case description

**Figure 1:**
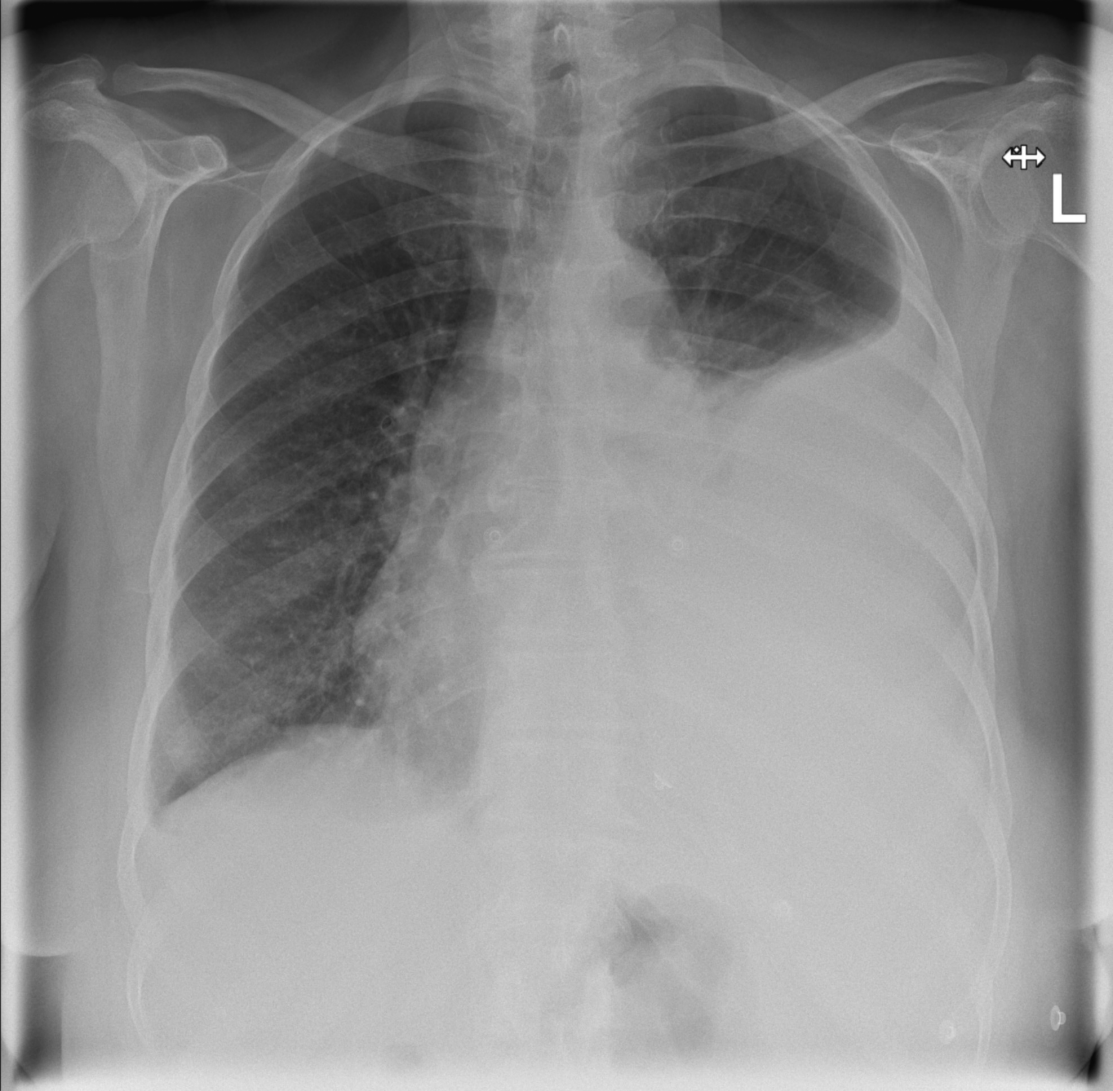
Initial chest x-ray with left pleural effusion.

A 73-year-old, fully conscious woman presented to our emergency department (Martha-Maria-Hospital in Dölau/Halle, Germany) due to dyspnea for the past 3 days. Her general condition was stable, and she denied any pain. At the time of presentation, she was an ex-smoker (cum. 10 PY). No cardiopulmonary events were recorded up to that time, and she denied taking anticoagulant medication. Physical examination revealed decreased breath sounds on the left side and no abnormalities on the right side. Her hemoglobin level was 7.0 mmol/L (reference range 7.1–9.9 mmol/L). The initial chest x-ray (see Figure 1) showed a left pleural effusion, which was managed by placing a 14 French chest tube in the left pleural cavity using the Seldinger technique. A total of 2,000 ml of bloody effusion fluid was fractionally drained. The patient was admitted to the pulmonary unit for further diagnosis and therapy (Table 1).

**Table 1:** Showing a timeline with relevant data from the episode of care.

Timeline of symptoms, investigation and treatment	
2022	Dyspnea since 3 days
	Chest x-ray: left pleural effusion
	Diagnosis of unilateral hemothorax (chest tube installation)
1 week later	1. VATS (left side) and biopsy (without malignancy)
2 weeks later	Right pleura with persisting bloody effusion
	2. VATS (right side) and biopsy (without malignancy)
1 week later	Thoracotomy, biopsy and tamponade (diagnosis of epithelioid angiosarcoma of the pleura)
	Several re-thoracotomies and thoracostomy because of recurrent bleeding
	prolonged ICU treatment
1 week later	Best supportive care at palliative unit
	CT scan: multiple cerebral metastases

### Diagnostic assessment, details on the therapeutic intervention, follow-up, and outcomes, as specified in the CARE guidelines

A contrast-enhanced computed tomography (CT) scan of the chest revealed persistent left pleural effusion, which was classified as serous to slightly hemorrhagic by density. There was no evidence of tumor formation, infiltration or pathologically enlarged thoracic lymph nodes. Bronchoscopy was unremarkable. Endobronchial ultrasound (EBUS) biopsy of the thoracic lymph nodes revealed squamous metaplasia. Cytology of the pleural effusion fluid revealed no malignant cells. Two erythrocyte concentrates were administered, resulting in a sufficient increase in hemoglobin. In the absence of malignant findings, the cause of the hemothorax was considered to be traumatic. Iron deficiency anemia was diagnosed, and oral iron supplementation was initiated.

**Figure 2:**
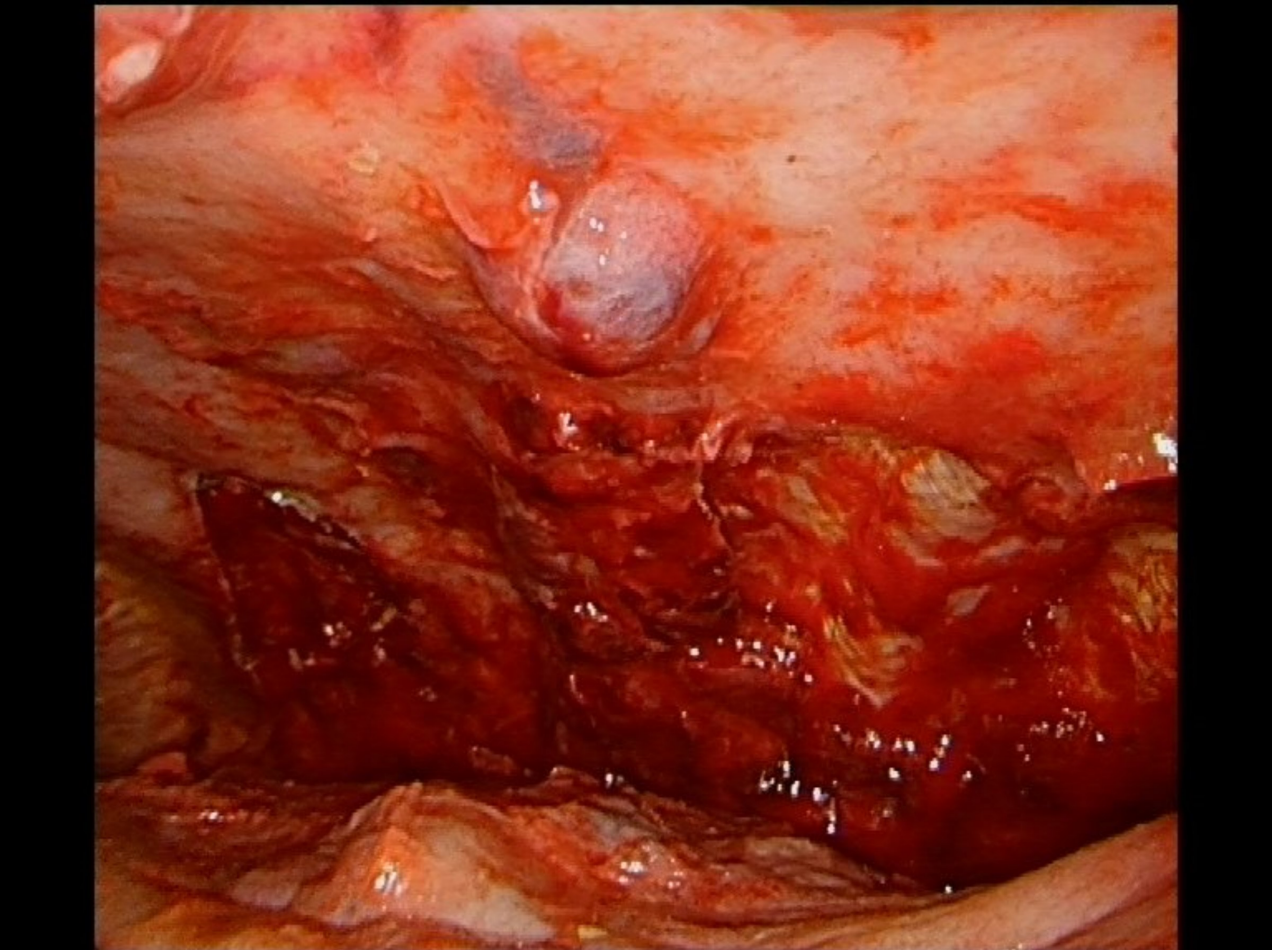
Image of the 1st video-assisted thoracoscopic surgery (VATS) after pleural biopsy showing abnormalities in the parietal pleura.

**Figure 3:**
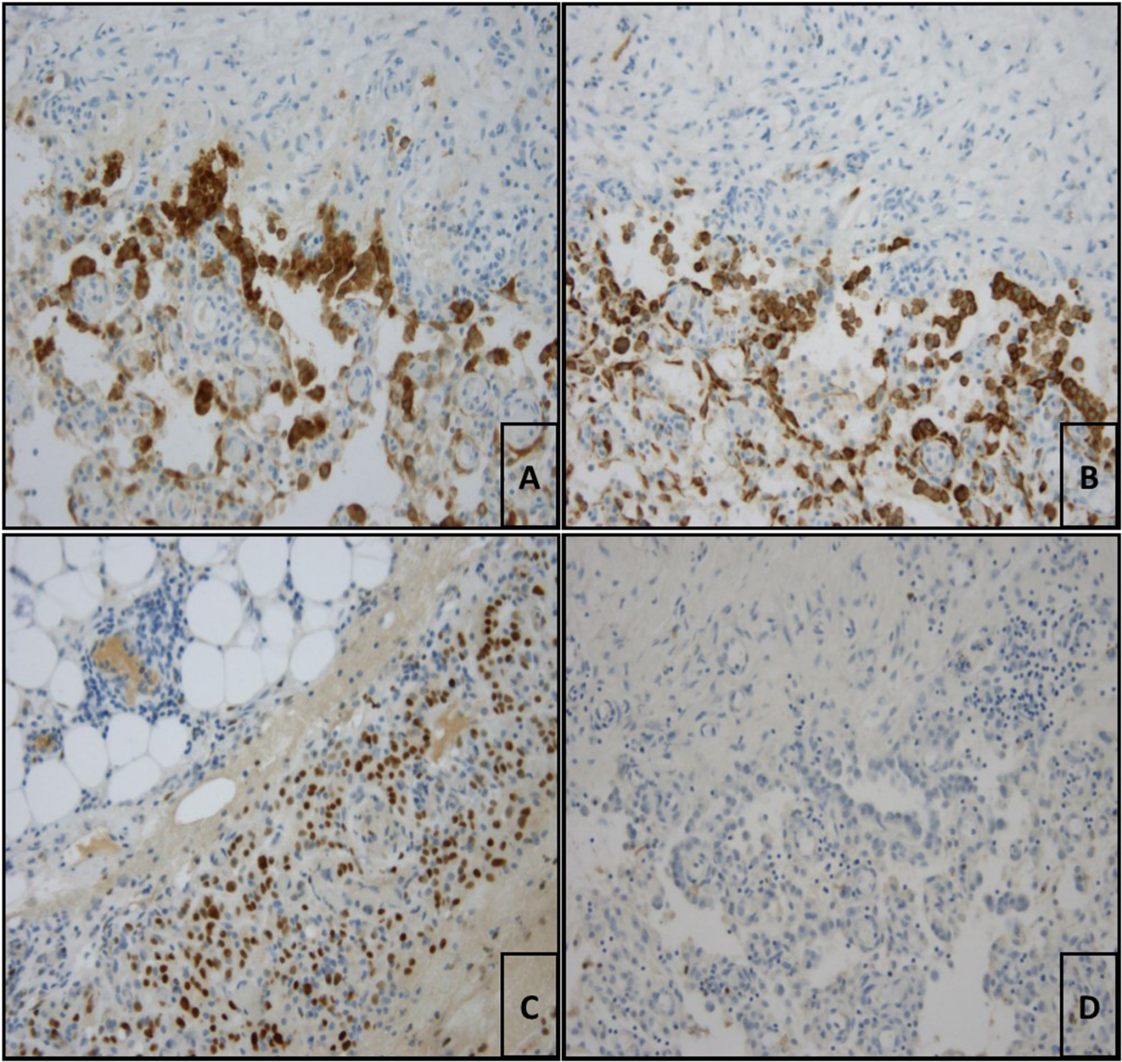
(**A**) Pleura visceralis with evidence of reactive proliferated mesothelia which are cytoplasmically labelled with antibodies against calretinin (calretinin (ZM85), 1:150 dilution, ZETA corporation, monospecific mouse-AK, IgG1/k; 200× magnification). (**B**) Pleura viszeralis showing proliferating mesothelia stained positive for panzytokeratin (Anti-Humanic Cytokeratin (pan), 1:75 dilution, Zytomed, AE1/AE3, mouse IgG1/k mouse IgG1/k; 200× magnification). (**C**) According to the immunstaining proliferating cells express WT-1 (WT49, 1:10 dilution, Leica; 200× magnification). (**D**) No immunohistological marking of the tumor cells with Ber-EP4 (EpCAM, Ber-EP4 (4), 1:100 dilution, DAKO; 200× magnification) which ruled out mesothelioma.

The patient was transferred to our thoracic surgery unit because of the persistence of a bloody pleural effusion. We performed video-assisted thoracoscopic surgery (VATS). Lesions of the parietal pleura were subsequently diagnosed (Figure 2). The pleura was intact, thickened and irregular. There was no active bleeding. The surgeon initially thought it was mesothelioma because of the parietal pleural manifestation, although the macroscopic impression was different. Several pleural samples were taken (2 samples, maximum size 48 cm²). Due to evidence of persistent bloody pleural effusion, a double chest drain was used. The pathological findings showed no evidence of malignancy, only a hemothorax with siderin deposits and granulation tissue. However, mesothelioma was ruled out by immunohistochemical analysis. Histopathological examination revealed strong nuclear BAP expression in the proliferated MSLN cultures. After incubation with an antibody against Ber-EP4 (EpCAM), carcinosis was excluded. The proliferating cells expressed Pan-Cytokeratin and WT-1 as well as focally weak calretinin. The immunohistological findings were suggestive of reactive proliferation, and neoplastic proliferation was consequently excluded (Figure 3). The chest tube was removed on the 3rd postoperative day, and the patient was discharged to the pulmonary department on the 5th postoperative day.

Surprisingly, the patient developed a right pleural effusion, which was also managed with a chest tube. Due to the persistence of the pleural effusion and the need for erythrocyte transfusion, VATS, which was performed by the same surgeon, was also indicated for the right hemothorax. The findings on the right side were almost identical to those on the left side. Multiple biopsies (3 samples, maximum size 12 cm²) were taken, the hemothorax was treated with a double chest tube, and the patient was transferred to our ICU. Unfortunately, the histopathological examination was inconclusive due to incorrect selection of antigens. However, due to increasing hemorrhagic effusion through the chest tube, surgical revision was mandatory. The bleeding was managed by right-sided thoracotomy, which allowed tamponade and extensive biopsy. The recurrent bleeding eventually led to the creation of a thoracostomy to facilitate repeated tamponade changes.

**Figure 4:**
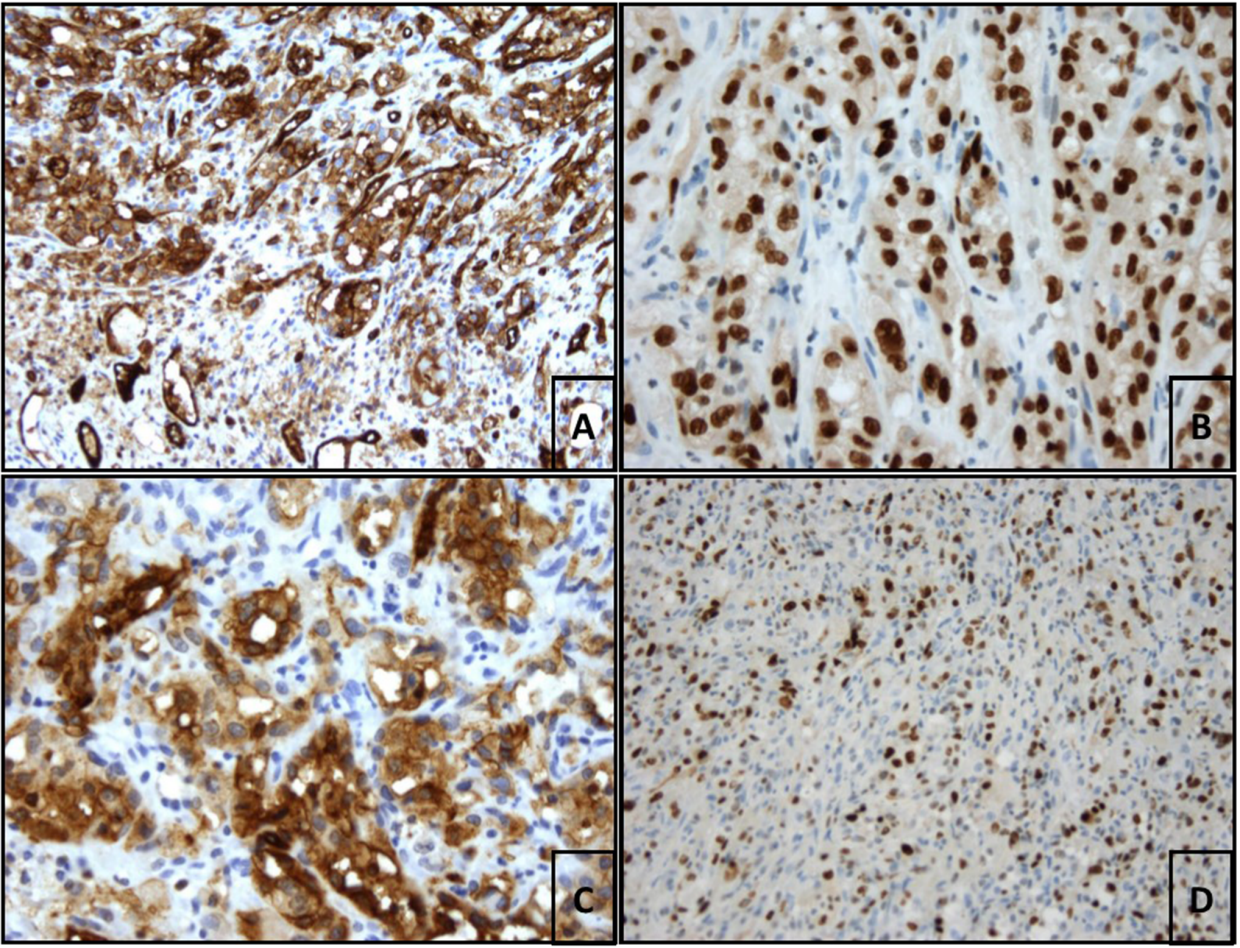
(**A**) Benign CD31-positive endothelia in the lower third of the image. In the upper 2 thirds of the image strongly CD31-positive epitheloid endothelial cells of the angiosarcoma (CD31 (JC70A), ready-to-use, Leica; 200× magnification. (**B**) Strong nuclear ERG staining of epitheloid tumor cells (EP111, ready-to-use, DAKO, 400× magnification). (**C**) Strong diffuse cytoplasmic staining of the neoplastic cells with antibodies against D2-40 (podoplanin, D2-40 (7), 1:50 delution, DAKO; 400× magnification). (**D**) Focal Ki67-proliferation rate of 80% (SP6, 1:100 delution, rabit IgG, Zytomed; 200× magnification).

An additional pathological report with further immunohistochemical staining for cluster of differentiation (CD) revealed the following results: massively proliferating epithelioid cells strongly positive for Erythroblast-transformation-specific Related Gene (ERG) and CD31. CD34 staining was negative. The neoplastic cells coexpressed D2-40 (podoplanin) and Ki67, with a proliferation rate of 80%. The final diagnosis was pleural manifestation of epithelioid angiosarcoma (Figure 4).

Due to the poor prognosis and general condition with a Karnosky score of 30%, the patient was finally transferred to our palliative care unit. A CT scan of the head revealed multiple cerebral metastases. The patient was discharged at the request of her family.

## Discussion

According to Morgan et al., “*spontaneous hemothorax is defined as a pleural fluid hematocrit greater than 50% of the peripheral blood hematocrit and the absence of natural or iatrogenic trauma to the lung or pleural space”* (10). However, the most common etiologies of hemathorax are traumatic, iatrogenic and coagulopathy (11). Cancer rarely causes true spontaneous hemathorax. However, bloody effusion is often associated with malignancy.

Rare cancers, such as hemangioendothelioma and hemangiosarcoma of the pleura or lung (12); schwannoma (13); primitive neuroectodermal, hepatocellular, vascular and germ cell tumors; primary lung cancer; mesothelioma; and malignancies leading to extramedullary hematopoiesis, are associated with spontaneous hematomas (10). Nonmalignant causes include endometriosis (14); neurofibromatosis type 1, also known as Recklinghausen's disease (15); and vascular events such as ruptured aortic aneurysm, pulmonary infarction from pulmonary embolism, necrotizing lung infection and hemopneumothorax (10). In addition, when spontaneous hemothorax occurs bilaterally, other differential diagnoses are more likely than when it occurs unilaterally.

Pleural epithelioid hemangiosarcoma has no specific clinical or imaging manifestations, so the diagnosis is based on histological findings, including immunohistochemical staining. It should be differentiated from adenocarcinoma, hemangioendothelioma, mesothelioma and pulmonary epithelioid hemangiosarcoma (12). Other authors have noted the difficulties in differentiating malignant mesothelioma from epithelioid hemangiosarcoma (7, 9) or even benign diagnoses (8).

As J. W. Goethe said, “*You only see what you already know”*. Therefore, physicians treating spontaneous hemothorax need to have broad knowledge of the possible, sometimes rare, etiologies. Preoperative investigations revealed no evidence of malignancy. Due to the persistence of the pleural effusion, the patient underwent surgery by an experienced thoracic surgeon who questioned the commonly suspected diagnosis of malignant mesothelioma because of lesions of the parietal pleura. This case highlights the need for appropriate communication between surgeons and pathologists. Even when biopsies are suitable for diagnosis, misdiagnosis can occur due to misinterpretation of the clinical course and misunderstanding of tumor localization and cell morphology, leading to inappropriate immunohistochemical staining programs. The cell morphology of angiosarcoma is diverse. Tumor cells may mimic fibroblasts or epithelial cells, making differential diagnosis difficult (7). According to the literature, pleural epithelioid angiosarcoma tumor cells are often positive for CD31 (5–7, 16, 17), vimentin (5–7, 16) and WT1 (17, 7). Analysis of CD34 may be positive (5, 6) or negative (14). The results published by Roh et al. showed weak positivity for factor VIII (5). Other authors published immunohistochemical analysis with negative factor VIII staining (16). Our staining was strongly positive for CD31, negative for CD34 and positive for D2-40 (podoplanin). The analysis published by Durani et al. was also positive for D2-40 (17). According to Sullivan et al., ERG staining is highly suitable for the cytological diagnosis of angiosarcoma (18).

## Conclusion

Pleural angiosarcoma should be considered in the differential diagnosis of recurrent pleural effusion of unknown etiology. An excisional biopsy is required to rule out the diagnosis. However, if the clinical course and intraoperative findings are not consistent with the histopathological findings, the diagnosis must be questioned, and further immunohistochemical staining is mandatory.

## Data Availability

The original contributions presented in the study are included in the article/Supplementary Material, and further inquiries can be directed to the corresponding author.
